# Frequency of HTLV-1 seroconversion between pregnancies in Nagasaki, Japan, 2011–2018

**DOI:** 10.3389/fmicb.2022.1036955

**Published:** 2022-11-15

**Authors:** Nahoko Komatsu, Masako Iwanaga, Yuri Hasegawa, Shoko Miura, Naoki Fuchi, Hiroyuki Moriuchi, Katsunori Yanagihara, Kiyonori Miura

**Affiliations:** ^1^Department of Obstetrics and Gynecology, Nagasaki University Graduate School of Biomedical Sciences, Nagasaki, Japan; ^2^Department of Clinical Epidemiology, Nagasaki University Graduate School of Biomedical Sciences, Nagasaki, Japan; ^3^Department of Pediatrics, Nagasaki University Graduate School of Biomedical Sciences, Nagasaki, Japan; ^4^Department of Laboratory Medicine, Nagasaki University Graduate School of Biomedical Sciences, Nagasaki, Japan

**Keywords:** HTLV-1, pregnant woman, carrier, screening test, seroconversion, horizontal transmission, proviral load, adult T-cell leukemia-lymphoma

## Abstract

**Background:**

Human T-cell leukemia virus type-1 (HTLV-1) is transmitted vertically from an infected mother to her child *via* breastfeeding during infancy or horizontally *via* sexual contact. However, little information is available on the HTLV-1 seroconversion rate in pregnant mothers and the impact of new HTLV-1 infection on mothers and babies during the perinatal period.

**Methods:**

From the database of a prefecture-wide antenatal adult T-cell leukemia prevention program in Nagasaki, Japan, we extracted data on 57,323 pregnant women who were screened for anti-HTLV-1 antibody during 2011–2018. Data on the 16,863 subjects whose HTLV-1 proviral load (PVL) was measured more than twice were included in our analyses.

**Results:**

In total, 133 (0.79%) pregnant women were HTLV-1-positive during their first pregnancy and nine (0.05%) seroconverted before or during subsequent pregnancies (between pregnancies). The median PVL (per 100 peripheral blood mononuclear cells) was significantly lower in the seroconverted mothers (0.10%) than in the initially seropositive mothers (0.15%). A repeated measures correlation analysis for the individual PVLs of the HTLV-1-positive pregnant women showed that PVL increased with parity number (*rrm* = 0.25) with no perinatal problems.

**Conclusion:**

The HTLV-1 seroconversion rate between pregnancies was 0.05%, and their HTLV-1 PVL increased annually but no perinatal problems were noted.

## Introduction

Human T-cell leukemia virus-1 (HTLV-1) is a retrovirus that causes adult T-cell leukemia (ATL), HTLV-1-associated myelopathy/tropical spastic paraparesis (HAM/TSP), and a variety of HTLV-1-associated inflammatory disorders (Watanabe, 1997). Globally, HTLV-1 infects at least 5–10 million people (Gessain and Cassar, 2012), particularly in southwest Japan, Brazil, the Caribbean islands, Central and South America, sub-Saharan Africa, and central Australia (Gessain and Cassar, 2012; World Health Organization, 2020). HTLV-1 infects mainly CD4-positive T-cells and is known to transmit from person-to-person *via* cell-to-cell contact between HTLV-1-infected cells and uninfected cells through three modes (Bangham, 2003; Matsuoka and Jeang, 2007; Pique and Jones, 2012): vertical mother-to-child transmission from HTLV-1-infected mothers *via* breastfeeding (Hino et al., 1985), horizontal transmission from HTLV-1-infected partners *via* sexual intercourse (Stuver et al., 1993; Kaplan et al., 1996; Roucoux et al., 2005; Satake et al., 2016; Paiva et al., 2017), and iatrogenic transmission *via* HTLV-1-infected donated blood or organs. However, the risk of iatrogenic transmission through transfusion can be reduced by performing leukoreduction prior to transfusion (Okochi et al., 1984; Armstrong et al., 2012).

**Figure 1:**
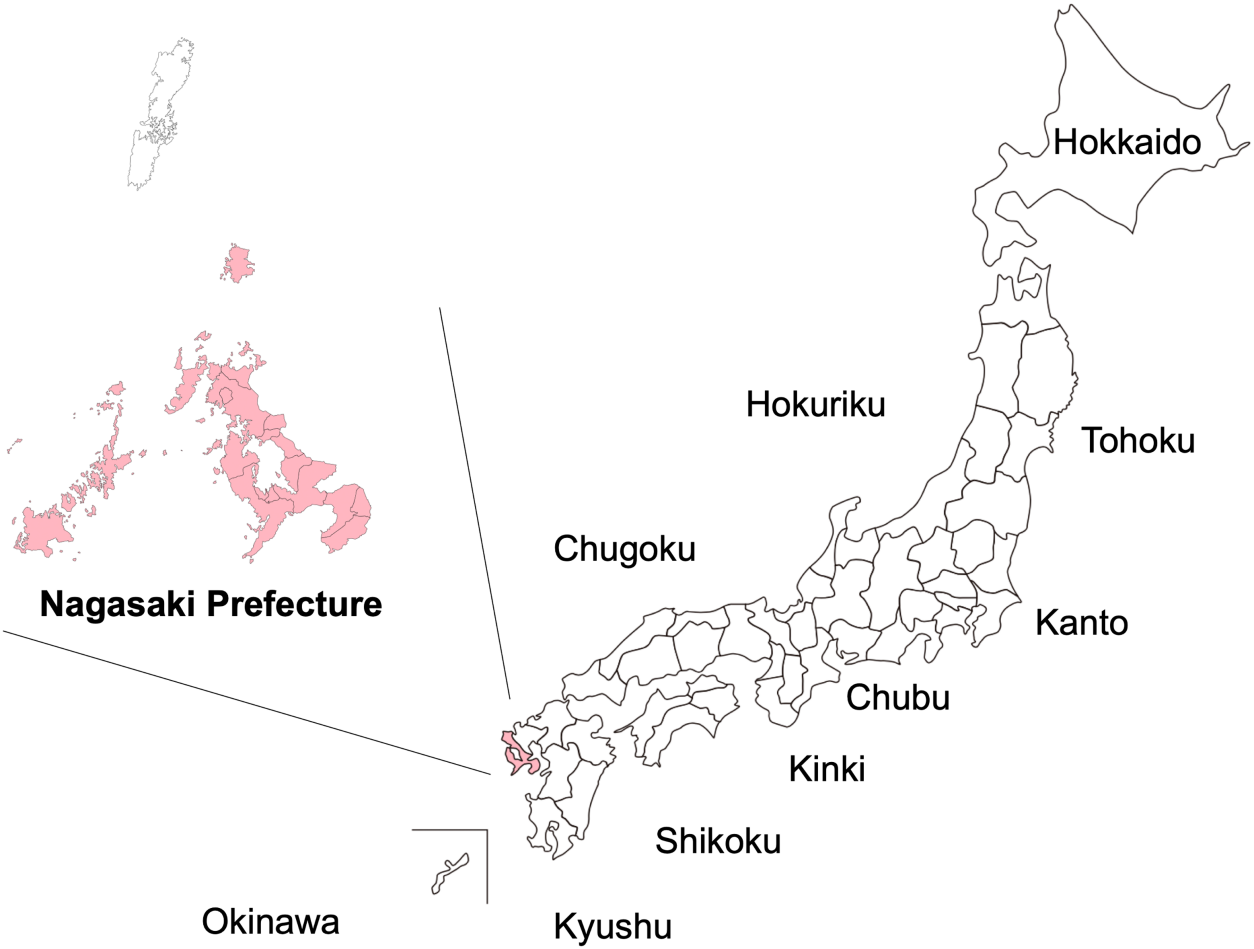
Location of the study area in Nagasaki Prefecture, Japan. The present study was carried out in Nagasaki Prefecture, which is located in the most western part of Japan. Only the part of Nagasaki Prefecture colored pink is included in this study. (Tsushima islands are not included).

The first scientific evidence of vertical transmission from a HTLV-1-infected mother to their infant *via* prolonged breastfeeding by the infected mother was presented in 1985 by researchers from Nagasaki University, located in southwest Japan (Hino et al., 1985; Figure 1). They presented the following findings: (1) the first nationwide survey of HTLV-1 infection in Japan identified Nagasaki Prefecture as one of the regions with the highest prevalence of HTLV-1-infected individuals (Tajima et al., 1982); (2) HTLV-1-infected cells are present in breastmilk from HTLV-1-carrier mothers, and infection through breastfeeding is associated with a higher probability of developing ATL (Kinoshita et al., 1984); and (3) experimentally, HTLV-1 can be transmitted *via* intraoral inoculation among common marmosets (Kinoshita et al., 1985; Yamanouchi et al., 1985). Soon after, a prefecture-wide, multidisciplinary, antenatal intervention program, known as the “ATL Prevention Program (APP),” was established in 1987 in Nagasaki (APP Nagasaki; Hino et al., 1987) through collaboration with virologists, obstetric gynecologists, hematologists, pediatricians, and clinical laboratory scientists at Nagasaki University, obstetric gynecologists throughout Nagasaki Prefecture, and public health officers of the Maternal and Child Health Division (MCHD) of the Nagasaki Prefectural Government. The purpose of APP Nagasaki was to reduce the vertical transmission of HTLV-1 *via* breastfeeding and to reduce the future development of HTLV-1-associated diseases through systematic screening for anti-HTLV-1 antibodies in pregnant women and the avoidance of breastfeeding in identified HTLV-1-infected mothers, in Nagasaki Prefecture.

As previously reported, APP Nagasaki led to a marked reduction in mother-to-child HTLV-1 transmission rates from 20.5% before the APP began to 2.4% after its implementation (Hino et al., 1997; Katamine, 1999; Hino, 2011; Moriuchi et al., 2013; Miura and Masuzaki, 2017). Other intervention programs similar to APP Nagasaki have been implemented in several areas endemic for HTLV-1 in Japan (Takahashi et al., 1991; Kashiwagi et al., 2004). However, HTLV-1 infection is currently widespread across geographical regions (Watanabe, 2011; Satake et al., 2012), and the number of HTLV-1 carriers in the metropolitan areas in Japan has increased. This situation probably reflects the migration of individuals from HTLV-1-endemic areas (Watanabe, 2011). A nationwide antenatal screening program for HTLV-1 was introduced in Japan in 2011 (Itabashi et al., 2020). Currently, it is estimated that each year ~1,600 HTLV-1-infected pregnant mothers in Japan (almost 0.16% of all pregnant mothers in Japan; Suzuki et al., 2014) are recommended to refrain from breastfeeding to avoid mother-to-child HTLV-1 transmission by methods similar to that of APP Nagasaki.

Recently, researchers from APP Nagasaki reported that the HTLV-1 proviral load (PVL), induced regulatory T-cell population, and soluble interleukin-2 receptor (sIL-2R) levels of HTLV-1-infected pregnant mothers remained stable during pregnancy but became elevated after delivery (Fuchi et al., 2018). However, many questions regarding the impact of HTLV-1 infection on pregnant mothers remain unanswered. For example, the number of pregnant mothers who seroconvert from HTLV-1 negative to positive between pregnancies, how the HTLV-1 PVL changes in HTLV-1-infected pregnant mothers through delivery (between pregnancies), and the specific risks to pregnancy outcomes for HTLV-1-infected and HTLV-1-seroconverted mothers are not currently known.

The present study therefore aimed to assess the HTLV-1 seroconversion status in multiparous mothers who participated in APP Nagasaki, the individual changes of the HTLV-1 PVL between pregnancies in HTLV-1-positive mothers, and the impact of HTLV-1 positivity on pregnancy outcomes.

## Materials and methods

### Study area and database

From its initiation in July 1987 to April 2018, the APP Nagasaki database included information on ~330,000 pregnant mothers, from almost all areas of Nagasaki Prefecture, who were tested for HTLV-1 infection (Figure 1). The database is managed by the Department of Obstetrics–Gynecology at Nagasaki University Hospital (Miura and Masuzaki, 2017).

### Protocol employed for APP Nagasaki

**Figure 2:**
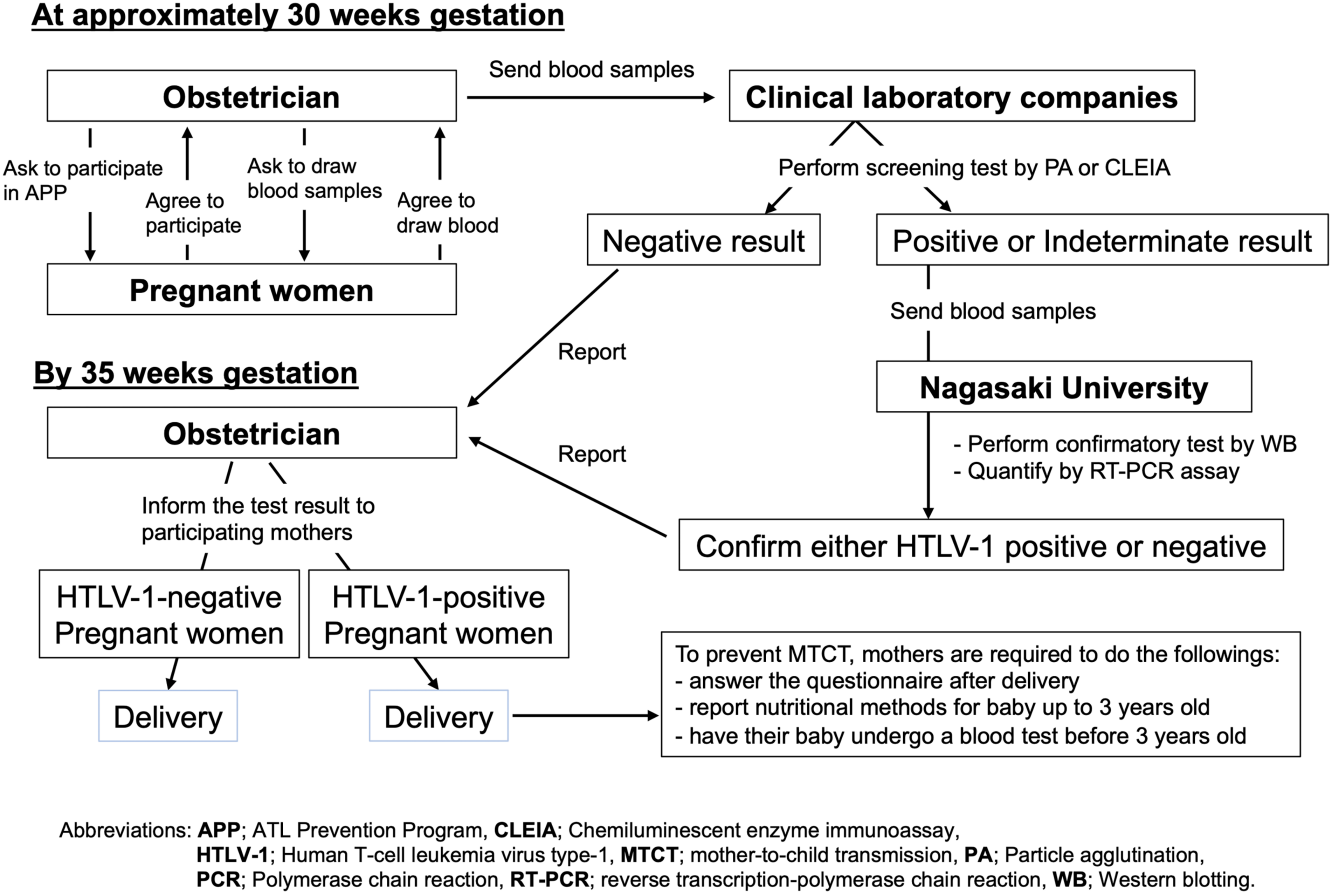
Study protocol of the APP Nagasaki project. By 30 weeks of gestation, blood samples were obtained from the pregnant mothers who participated in APP Nagasaki to screen for anti-HTLV-1 antibody. Negative test results were reported directly to the respective obstetrician. For positive or indeterminate test results, confirmatory tests were performed. All of the test results were scheduled to be returned to the respective obstetricians by 35 weeks of gestation.

Figure 2 shows the protocol employed for APP Nagasaki (Miura and Masuzaki, 2017). Briefly, any pregnant mothers who were ~30 weeks of gestation and living in most areas of Nagasaki Prefecture were asked to participate in APP Nagasaki when visiting their obstetric gynecologists. After providing consent to participate in the project, each pregnant mother was required to provide a blood sample for an anti-HTLV-1 antibody test. From 2011 onwards, such blood samples were sent to one of five commercial clinical laboratory companies (see Acknowledgment) where a screening test for anti-HTLV-1 antibody was performed using either a particle agglutination (PA) assay, a chemiluminescent immunoassay (CLIA), or a chemiluminescent enzyme immunoassay (CLEIA). The definition of “HTLV-1-positive” as a result of the screening test is a titer of 8 or higher in the PA assay or a cutoff index (COI) of ≥1.0 in the CLEIA or CLIA, in accordance with the manufacturer’s criteria (Fujirebio, Japan).

All of the negative screening test results from the clinical laboratory companies were sent back to the respective obstetric gynecologists, for them to inform the respective pregnant mothers. From 2011 onward, in the case of a positive or suspected positive screening test result, the residual blood sample was forwarded to the APP Nagasaki office at Nagasaki University Hospital where a confirmatory test was performed by western blotting and the HTLV-1 PVL was quantified by a real-time polymerase chain reaction (RT-PCR) assay in the Department of Laboratory Medicine. The final test results were reported to the respective obstetric gynecologists, who then informed the respective mothers by 35 weeks of gestation (Figure 2). The obstetric gynecologists also asked their public health nurses to explain to HTLV-1-positive pregnant mothers how to prevent HTLV-1 mother-to-child transmission, preferentially through exclusive bottle-feeding or alternatively by freeze–thawing breast milk or only short-term breastfeeding for no more than 3 months. According to the APP Nagasaki report, the majority of HTLV-1-positive pregnant mothers chose the first choice option of exclusive bottle-feeding to prevent mother-to-child transmission (Miura and Masuzaki, 2017).

### Quantification of the HTLV-1 PVL

In APP Nagasaki, a quantitative RT-PCR assay was routinely performed from 2011 onward to quantify the HTLV-1 PVL in peripheral blood mononuclear cells (PBMCs) from anti-HTLV-1 antibody-positive pregnant mothers and those with indeterminate western blotting results. The details of the method used to quantify the HTLV-1 PVL have been previously published (Kamihira et al., 2003; Sasaki et al., 2010), and the resulting value was expressed as the copy number per 100 PBMCs. All measurements were performed by the Department of Laboratory Medicine at Nagasaki University Hospital. Coefficient variation (CV) of HTLV-1 quantitative PCR test in Nagasaki university hospital was 13.3% (Kuramitsu et al., 2015).

### Questionnaires for HTLV-1-positive mothers and their doctors after delivery

Within 1 week of delivery, each of the HTLV-1-positive mothers who participated in APP Nagasaki was required to answer questions about their birthplace, pregnancy history, the feeding method chosen by their own mother (i.e., the grandmother) during their infancy, and the feeding method that they chose for their own baby. Their obstetric gynecologist was also required to answer questions regarding pregnancy/delivery complications, the health condition of the baby, and the medical history of the pregnant mother (Supplementary material S1).

### Data extraction for analysis

The dataset used for analysis in this study was extracted from the APP Nagasaki database after obtaining approval from the Ethics Committee of the Institutional Review Board of Nagasaki University Hospital, Nagasaki, Japan (approval number: 20051810, September 2019). The APP Nagasaki was initiated in 1987, and modern techniques to measure the HTLV-1 PVL using an RT-PCR assay have been applied to participating mothers only since 2011 (Kamihira et al., 2003; Sasaki et al., 2010). Therefore, for the present analysis, we limited our data extraction to the years 2011–2018.

### Statistical analysis

We analyzed the available data on pregnant mothers who were tested for HTLV-1 infection at least twice during pregnancy. The demographic data were summarized with standard descriptive statistics, such as frequencies (percentages) for categorical variables, and median (min–max) or median (interquartile range, IQR) values for continuous variables. The pregnant mother age at enrollment in the APP was treated as a continuous variable or categorized into four groups (<25, 26–30, 31–35, and >36 years old). The method of feeding that the pregnant mothers themselves received from their own mothers was categorized into three groups: long-term breastfeeding (6 months or longer), short-term breastfeeding (shorter than 6 months), and formula feeding alone. The mothers were also categorized into subgroups based on their history of gravidity (the number of pregnancies), parity (the number of live births), and divorce. Continuous variables were compared using Wilcoxon rank-sum tests. Categorical variables were compared using the Chi-squared test or Fisher’s exact test, as appropriate. All of the basic statistical analyses were performed using OpenEpi Statistical Software version 3.01 (http://www.openepi.com, Atlanta, GA, United States; Sullivan and Dean, 2009) and R package version 3.4.2 (R-Project for Statistical Computing, https://CRAN.R-project.org). To analyze changes over time in the HTLV-1 PVL by age and by parity of the HTLV-1-positive mothers, we conducted repeated measures correlation (rmcorr) analyses using the R package version 0.3.0 (R-Project for Statistical Computing, https://CRAN.R-project.org; Bakdash and Marusich, 2017) and calculated the correlation coefficient for repeated measures (*rrm*). All statistical tests were performed at the two-tailed 5% level of significance.

## Results

### HTLV-1 seropositive status

**Figure 3:**
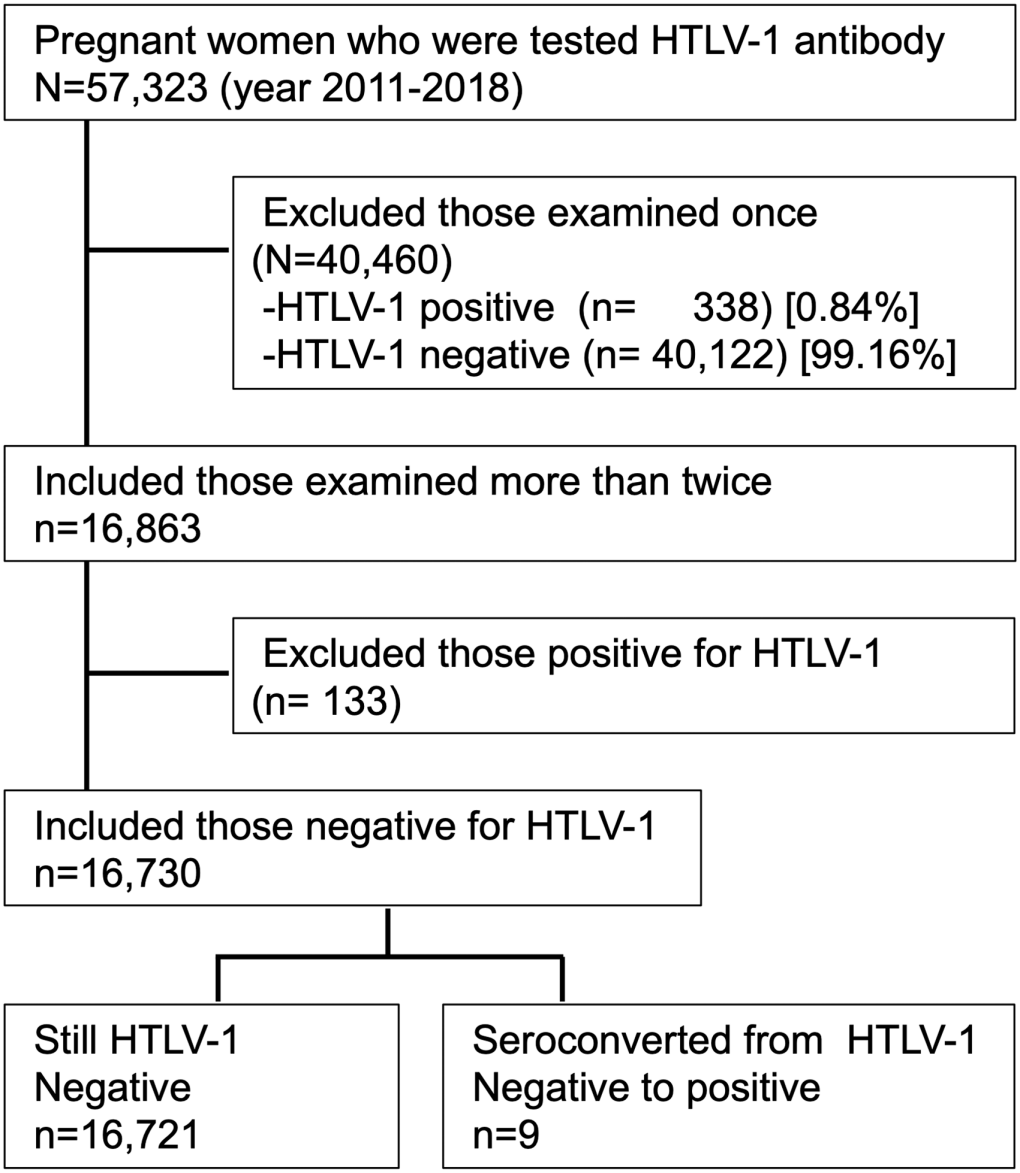
Study flowchart for the present analysis. Of the 57,323 pregnant mothers who were screened for anti-HTLV-1 antibody during 2011–2018, the 16,863 mothers who were examined more than twice were included in the study. Of those included, the 16,730 mothers who tested negative for anti-HTLV-1 antibody were included in the analysis. Nine of these women later seroconverted to HTLV-1-positive.

Figure 3 shows the flowchart for the present analysis. In the years 2011–2018, a total of 57,323 pregnant mothers were screened for anti-HTLV-1 antibody, and 471 (seropositive rate of ~0.82%) were identified as being HTLV-1-positive. Among the 57,323 screened women, 40,460 (70.6%) were examined only once, and the remaining 16,863 (29.4%) were examined more than twice. Of those examined only once, 338 (0.84%) were HTLV-1-positive; of those examined more than twice, 133 (0.79%) were HTLV-1-positive. Among the 133 HTLV-1-positive pregnant mothers screened during their first pregnancy, 61 (45.9%) had already been diagnosed as asymptomatic HTLV-1 carriers in a hospital before their enrollment with APP Nagasaki, but the remaining 72 had no prior HTLV-1 diagnosis information. To date, there have been no reports about ATL, HAM/TSP, or other HTLV-1-associated diseases from their obstetric gynecologists.

### HTLV-1 seroconversion rate

During the period between January 2011 and December 2018, nine (0.054%) of the 16,730 pregnant mothers who were found to be negative for HTLV-1 during the initial screening subsequently seroconverted to HTLV-1 positive (Figure 3). Thus, the incidence rate of HTLV-1 seroconversion among HTLV-1-negative pregnant mothers was 17.5 (95% confidence interval: 11.4–26.0) per 100,000 person-years at follow-up.

### Background characteristics

Table 1 summarizes the characteristics of pregnant mothers who were already known to be HTLV-1 seropositive before their entry into the study (*n* = 133) and those of mothers who were HTLV-1 seronegative at study entry but later seroconverted to anti-HTLV-1 antibody-positive (*n* = 9). The median age at first entry to APP Nagasaki was 30 years old (range, 17–47 years old) in mothers of known HTLV-1-seropositive status and 31 years old (range, 23–38 years old) in HTLV-1-seroconverted mothers. Although there was no statistically significant difference in the age distribution between the two groups, approximately half of those already known to be HTLV-1-seropositive were in their late 20 s at their first pregnancy, whereas approximately half of the HTLV-1-seroconverted mothers were in their 30 s at their first pregnancy. There was also no significant difference in the proportions of mothers with a family history of ATL, a divorce history, or particular screening test values or in the proportion of western blot-positive or -indeterminate individuals between the two groups.

**Table 1:** The characteristics of HTLV-1-positive or HTLV-1-seroconverted pregnant women.

Characteristic	Mothers HTLV-1-positive at first pregnancy (*n* = 133)	Mothers seroconverted to HTLV-1-positive (*n* = 9)	*p*-Value
Age (year) at entry to APP Nagasaki			
Median (min, max), year	30 (17, 41)	31 (23, 38)	0.42
Age category, *n* (%) <25	19 (14.3)	2 (22.2)	
26–30	58 (43.6)	1 (11.1)	
31–35	44 (33.1)	5 (55.6)	
>36	12 (9.0)	1 (11.1)	
Nutrition method that participant mothers received from their mothers, *n* (%); [unknown = 22]			
Long-term breastfeeding	85 (63.9)	4 (44.4)	
Short-term breastfeeding	14 (10.5)	2 (22.2)	
Formula feeding	14 (10.5)	1 (11.1)	
Family history of ATL, *n* (%)			
Yes	9 (6.8)	1 (11.1)	
No	124 (93.2)	8 (88.9)	
Gravidity history, *n* (%); [unknown = 6]			
Once	61 (45.9)	0	
Twice	39 (29.3)	3 (15.8)	
Three or more	27 (20.3)	6 (31.6)	
Parity history, *n* (%); [unknown = 5]			
Once	76 (59.4)	0	
Twice	36 (28.1)	4 (44.4)	
Three or more	16 (12.5)	5 (55.6)	
Divorce history, *n* (%)			
Yes	10 (7.5)	1 (11.1)	
No	123 (92.5)	8 (88.9)	
Screening test values			
PA, titer	1,024 (16–8,192)	1,024 (16–8,193)	0.69
CLEIA, COI	34.4 (2.5–45)	34.4 (2.5–46)	0.47
WB test result, *n* (%); [unknown = 53]			
Positive	72 (90)	8 (88.9)	0.99
Indeterminate	8 (10)	1 (11.1)	
Quantified PVL, % per 1,000 PBMCs			
Median (min, max)	0.15 (0–7.05)	0.10 (0–1.09)	0.024

HTLV-1, human T-cell leukemia virus-1; APP, ATL Prevention Program; PA, particle agglutination; CLEIA, chemiluminescent enzyme immunoassay; COI, cutoff index; PVL, proviral load; PBMCs, peripheral blood mononuclear cells.

In contrast, there were significant differences between the two groups regarding the feeding method that the participating mothers themselves received from their mothers (i.e., the grandmothers), gravidity history, parity history, and HTLV-1 PVL. In brief, compared with the group of those already known to be HTLV-1-positive before pregnancy, the group of mothers that underwent HTLV-1 seroconversion between pregnancies had lower HTLV-1 PVL (0.15% vs. 0.10% per 100 PBMCs; *p* < 0.024). Regarding whether the mothers had known about the HTLV-1 infection status of their husband or sexual partner, only two pregnant mothers reported HTLV-1 infection in their partner; the rest of the study participants did not know their partner’s HTLV-1 infection status.

### Sequential changes in the HTLV-1 PVL in HTLV-1-positive pregnant mothers

**Figure 4:**
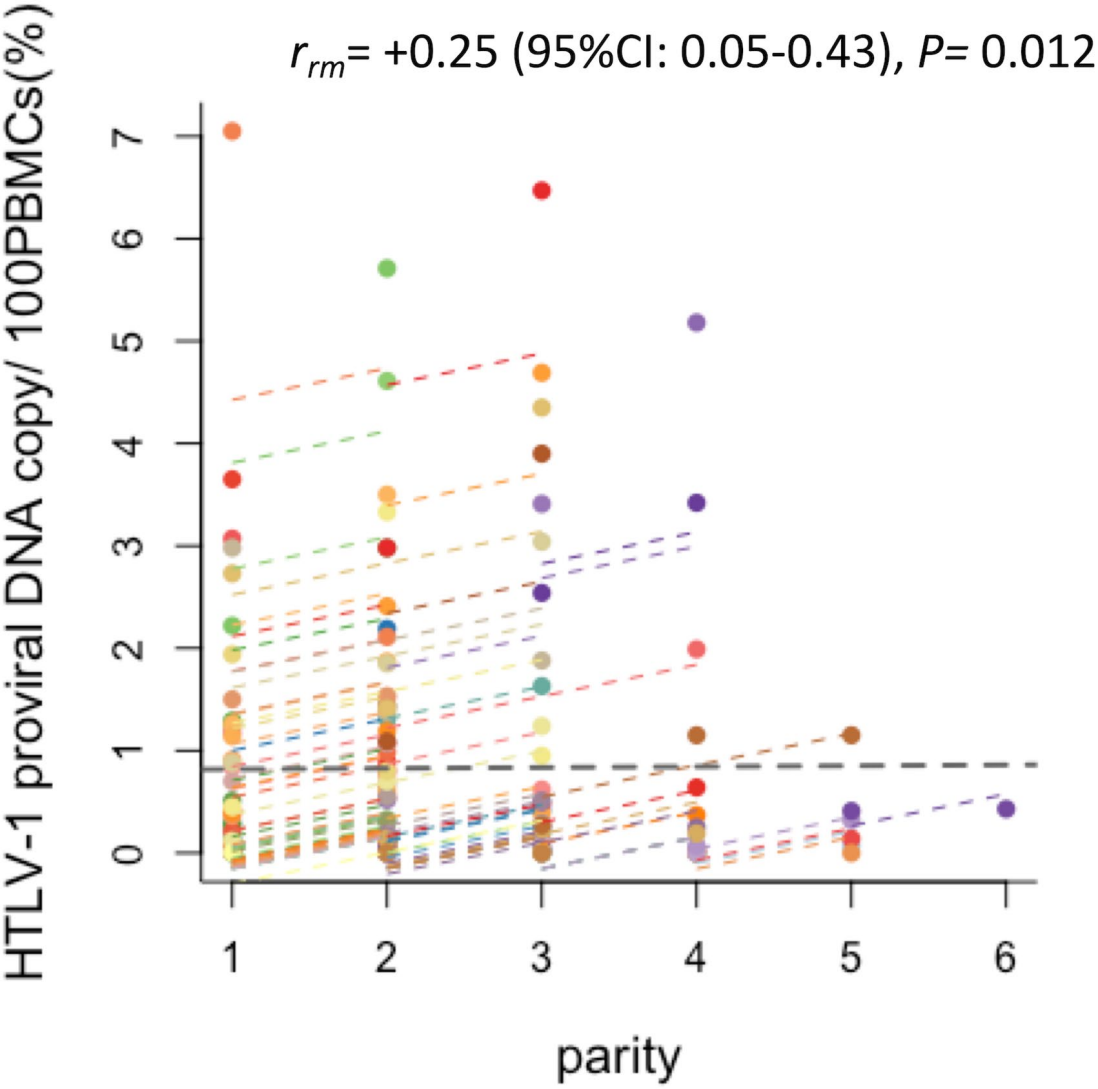
Changes in the HTLV-1 PVL of HTLV-1-positive pregnant women by parity. Plots of the correlation between the HTLV-1 PVL and parity of HTLV-1-positive pregnant women. Of the study participants, the 87 who were HTLV-1 seropositive and had their PVL measured more than twice were analyzed by repeated measures correlation (rmcorr). Each colored plot line shows the chronological change in HTLV-1 PVL of an individual HTLV-1-carrier pregnant woman. Positive correlations were found between increased HTLV-1 PVL and increased parity (*r* = 0.25, *p* = 0.012, 95% CI 0.05, 0.43).

An analysis of the HTLV-1-positive mothers revealed a statistically significant positive correlation between an increase in parity number and an increase in HTLV-1 PVL (*rrm* = +0.25, *p* = 0.012; Figure 4).

### Sequential changes in the HTLV-1 PVL In HTLV-1-seroconverted pregnant mothers

Among the nine HTLV-1-seroconverted pregnant mothers, there was only one mother (No. 2 in the Supplementary material S2) for whom the HTLV-1 PVL had been measured more than twice after HTLV-1 seroconversion. Her HTLV-1 PVL increased from 1.09 at her second delivery to 3.90 at her third delivery, which suggests that, even in HTLV-1-seroconverted pregnant mothers, the HTLV-1 PVL increased over time.

### Pregnancy outcomes

Table 2 summarizes the pregnancy outcomes of mothers of known HTLV-1-positive status and mothers that seroconverted to HTLV-1 between pregnancies. There was no statistically significant difference between the two groups in the frequency of any complications that occurred during pregnancy, the length of delivery time, or the sex distribution or birth weight of the babies.

**Table 2:** Pregnancy outcomes in mothers with known HTLV-1-positive status and mothers with HTLV-1 seroconversion during pregnancy.

Characteristic	Mothers HTLV-1-positive at first pregnancy (*n* = 133)	Mothers seroconverted to HTLV-1-positive (*n* = 9)	*p*-Value
No. of deliveries	265	10	−
Complications during pregnancy, *n* (%)			
No	222 (83.8%)	9 (90.0%)	0.67
Yes	43 (16.2%)	1 (10.0%)	
Length of delivery time, *n* (%)			
<34 weeks of gestation	222 (83.8%)	9 (90.0%)	0.37
34–36 weeks of gestation	11 (4.2%)	1 (10.0%)	
After 36 weeks of gestation	32 (12.1%)	0	
Sex of child, *n* (%)			
Unknown	7 (2.6%)	0	0.25
Male	130 (49.1%)	7 (70%)	
Female	128 (48.3%)	3 (30%)	
Birth weight, *n* (%)			
LFD	18 (6.8%)	1 (10.0%)	0.51
AFD	217 (81.9%)	9 (90.0%)	
HFD	30 (11.3%)	0	

LFD, light for dates; AFD, appropriate for dates; HFD, heavy for dates.

## Discussion

This is the first report on the HTLV-1-positive and -seroconversion status of pregnant mothers that participated in APP Nagasaki, and the first to report HTLV-1 PVL chronological data. Our main findings were that: (1) the proportion of those who underwent seroconversion from anti-HTLV-1 antibody negative to positive among pregnant mothers was 0.054%, with a seroconversion incidence rate of 17.5 per 100,000 person-years during the period 2011–2018, (2) HTLV-1 infection had no adverse effect on pregnancy outcomes in either the HTLV-1-infected or HTLV-1-seroconverted mothers; however, (3) the HTLV-1 PVL increased with increased parity number, and this showed a statistically significant positive correlation in both the HTLV-1-infected and the HTLV-1-seroconverted pregnant mothers.

The estimated incidence rate (17.5 per 100,000 person-years) of HTLV-1 seroconversion among pregnant mothers aged 17–41 years in this analysis was higher than that for female blood donors aged 16–49 years (2.03–8.54 per 100,000 person-years) in Kyushu-Okinawa, where HTLV-1 is endemic (Satake et al., 2016), despite the similar age range of these groups. A possible explanation for this difference might be the eligibility criteria for blood donation, because pregnant women or recently postpartum mothers are not eligible to donate blood in Japan. Instead, the estimated crude incidence rate in this analysis was close to the HTLV-1 seroconversion rate in female repeated blood donors aged over 50 years in Kyushu-Okinawa (11.8–17.0 per 100,000 person-years; Satake et al., 2016). One possible explanation for the HTLV-1 seroconversion rate similarity in these two groups might be related to unprotected sexual contact with HTLV-1-infected partners—the majority of pregnant mothers in our study did not know the HTLV-1 infection status of their partner.

Regarding the PVL in HTLV-1-positive pregnant mothers, we previously reported that the PVL was the highest during the postpartum period (i.e., the period just after delivery) as compared with that during pregnancy terms (i.e., the first, second, and third trimesters; Fuchi et al., 2018). The present study supported the previous report by performing time-sequential analyses of the individual PVL of both HTLV-1-positive and HTLV-1-seroconverted pregnant mothers. In other words, we hypothesize that the positive correlation between the number of deliveries and HTLV-1 PVL found in this study indicates that the elevated HTLV-1 PVL that occurs during the first postpartum period is maintained and then the HTLV-1 PVL increases further during the second postpartum period. Thus, the individual HTLV-1 PVL increased with increased parity number among multiparous women (Figure 4). Alternatively, this finding could suggest that these women are continuously exposed to HTLV-1 from their HTLV-1-positive partners (Stuver et al., 1993; Sullivan et al., 1993; Kaplan et al., 1996). Therefore, they require regular medical check-ups and monitoring of the HTLV-1 PVL for the early detection of HTLV-1-associated diseases, not only by obstetric gynecologists, but also by hematologists. This is primarily because it has been well-established that the higher the PVL in HTLV-1-infected asymptomatic carriers, the higher their risk of developing HTLV-1-associated diseases (Nagai et al., 1998; Iwanaga et al., 2010), but also because a number of case reports have indicated the development of ATL in HTLV-1-positive pregnant mothers with a high PVL (Ohba et al., 1988; Utsumi et al., 1996; Safdar et al., 2002; Amor et al., 2013; Motedayen Aval et al., 2020; Ramassamy et al., 2020). In APP Nagasaki, one asymptomatic HTLV-1-positive pregnant woman developed ATL (Fuchi et al., 2016).

We also report here, for the first time, that even in a HTLV-1-seroconverted mother, the PVL increased between her second and third pregnancies with her increase in age (the second case in Supplementary material S2). This finding supports the idea that the HTLV-1 PVL increases with parity (or age) even in HTLV-1-seroconverted pregnant mothers. Therefore, we recommend that all HTLV-1-positive women of reproductive age be checked regularly for their HTLV-1 PVL and HTLV-1-associated diseases.

Regarding pregnancy complications in HTLV-1-positive pregnant mothers, previous studies reported no difference in the degree of pregnancy complications between those who were positive and those who were negative for anti-HTLV-1 antibody (Kusuhara et al., 1987; Rosadas et al., 2019). In the present study, among the 10 deliveries from nine HTLV-1-seroconverted pregnant mothers, only one baby (10% of all deliveries) was born prematurely at 29 weeks of gestation. Although only one premature case occurred, and therefore the sample size was small, the proportion (10%) was greater than the national average for premature cases (5.6%–5.7%) in Japan during 2015–2018 (Vital Statistics of Japan, 2015–2018). Further large-scale analyses are required to determine whether the occurrence of preterm/premature birth/delivery/labor is higher in HTLV-1-infected and/or HTLV-1-seroconverted pregnant mothers than in uninfected individuals.

Regarding the babies born from HTLV-1-positive mothers, Kusuhara et al. reported that such babies may acquire their own anti-HTLV-1 antibodies by the age of 3 years and continue to acquire such antibodies until the age of 18 (Kusuhara et al., 1987). Therefore, the obstetric gynecologists participating in APP Nagasaki advised each of the HTLV-1-positive mothers to have their children tested for HTLV-1 antibodies when they reach 3 years of age. In APP Nagasaki, 10 children were born from HTLV-1-seroconverted mothers; of these, one baby girl was given artificial nutrition and tested HTLV-1-negative, while no information was available on the other nine babies.

The major limitation of this study is that the findings were based on a single area of Nagasaki Prefecture; therefore, the results may not apply to other regions of Japan. Another limitation is that our analyses were based only on healthy pregnant women; as such, information on pregnant mothers with possible HTLV-1-associated diseases was lacking. Furthermore, our method was not capable of identifying cases in which a pregnant mother is HTLV-1-negative at the screening date but seroconverts to positive after the screening date. Therefore, our results might be an underestimation. Furthermore, we have little information on the HTLV-1-infection status of the partners of the pregnant mothers. Taking these limitations into account, the actual number of pregnant mothers horizontally infected with HTLV-1 might be higher than what we observed in this study. Also, since our APP project is a kind of a public-health project to avoid mother-to-child HTLV-1-transmission rather than a research-oriented project, the accuracy of data collection may not be perfect and only peripheral blood test and HTLV-1 PVL are regularly measured. Therefore, even if we are willing to perform a multivariate analysis, it is possible to include only factors such as age and the number of deliveries. However, since aging and the number of deliveries are almost proportional, therefore it is difficult to perform a multivariate analysis as another limitation of our study.

In conclusion, this is the first report on the individual change in HTLV-1 PVL of HTLV-1-positive and HTLV-1-seroconverted pregnant mothers and the infection status of their babies in APP Nagasaki. Although there were no serious adverse effects in the HTLV-1-infected pregnant mothers and their babies, we found that the HTLV-1 PVL of the mothers increased with parity (or age), which suggests that HTLV-1-infected women of reproductive age are continuously exposed to HTLV-1 horizontally from their HTLV-1-infected partners. Therefore, further careful follow-up is needed for both HTLV-1-positive pregnant mothers and their babies.

## Data availability statement

The original contributions presented in the study are included in the article/Supplementary material, further inquiries can be directed to the corresponding author.

## Ethics statement

The studies involving human participants were reviewed and approved by the Ethics Committee of the Institutional Review Board of Nagasaki University Hospital, Nagasaki, Japan (approval number: 20051810, September 2019). The patients/participants provided their written informed consent to participate in this study.

## Author contributions

NK and NF analysis the data of HTLV-1 test among pregnant women. MI and KM organized the study protocol. NK, MI, and KM wrote the manuscript. YH, SM, NF, and KM collected the data of HTLV-1 tests among pregnant women. HM collected the data regarding Mother to Child transmission of HTLV-1. KY and KM performed the HTLV-1 screening test of pregnant women in Nagasaki. All authors contributed to the article and approved the submitted version.

## Funding

This work was partially supported by the Research Program (20fk0108124j0001, 21fk0108124j0002, 22fk0108124j0003) on Emerging and Re-emerging Infectious Diseases from the Japan Agency for Medical Research and Development (AMED) awarded to KM. The content of this publication is solely the responsibility of the authors and does not necessarily represent the official views of AMED.

## Conflict of interest

The authors declare that the research was conducted in the absence of any commercial or financial relationships that could be construed as a potential conflict of interest.

## Publisher’s note

All claims expressed in this article are solely those of the authors and do not necessarily represent those of their affiliated organizations, or those of the publisher, the editors and the reviewers. Any product that may be evaluated in this article, or claim that may be made by its manufacturer, is not guaranteed or endorsed by the publisher.
