# Supplementary material for: Frequency of HTLV-1 seroconversion between pregnancies in Nagasaki, Japan, 2011–2018
Source: Front Microbiol. 2022 Nov 15;13:1036955. doi: 10.3389/fmicb.2022.1036955 (PMC9705752; doi:10.3389/fmicb.2022.1036955)
Supplement: Supplementary file 1 [file Data_Sheet_1.docx]

Supplementary Material

**Supplementary Material (S-1, S-2)**

**Table S-1. Questionnaire items for HTLV-1-positive mothers and their doctors**

After delivery, all the HTLV-1-positive pregnant mothers and their obstetrics-gynecologists are required to answer to a set of questionnaire, which includes the following items.

| **(1)About pregnant women themselves** | - Date of birth / Birthplace / Nutritional methods which HTLV-1 carrier  - Pregnant women received at birth  - Family members who know that pregnant women are HTLV-1 carrier |
| --- | --- |
| **(2)About pregnancy** | -The number of gravidity and parity / Smoking or not during pregnancy  - Detail of pregnancy outcomes |
| **(3)About babies** | - Gestational week at delivery / Date of birth of babies/ Birth weight  - Sex / Delivery methods (including induction of labor)  - Nutritional method chosen by pregnant women |

**Table S-2. Sequential changes in HTLV-1 PVL in the HTLV-1-seroconversted pregnant mothers**

| **Case** | **Age**  **at delivery** | **Parity*** | **Screening test**  **result** | **PVL copy numbers**  **per 100 PBMCs** | **Baby’s**  **Birth weight**  **(g)** | **Pregnancy**  **complications** |
| --- | --- | --- | --- | --- | --- | --- |
| 1 | 28 | 2 | − | BT | 2820 | None |
|  | 31 | 3 | + | 0.02 | 2810 | None |
| 2 | 27 | 1 | − | n.m. | Unknown | Unknown |
|  | 30 | 2 | + | 1.09 | 2802 | None |
|  | 31 | 3 | + | 3.90 | 3336 | Gestational diabetes |
| 3 | 32 | 1 | − | n.m. | Unknown | Unknown |
|  | 35 | 3 | + | 0 | 2690 | None |
| 4 | 29 | 1 | − | n.m. | Unknown | Unknown |
|  | 31 | 2 | + | 0.02 | 3500 | None |
| 5 | 32 | 1 | − | n.m. | Unknown | Unknown |
|  | 35 | 2 | + | 0.52 | 3416 | None |
| 6 | 26 | 1 | − | n.m. | Unknown | Unknown |
|  | 31 | 4 | + | 0.01 | 3408 | None |
| 7 | 19 | 1 | − | n.m. | Unknown | Unknown |
|  | 21 | 2 | − | n.m. | Unknown | Unknown |
|  | 23 | 3 | + | 0 | 1854 | Premature birth (33w) |
| 8 | 33 | 1 | − | n.m. | Unknown | Unknown |
|  | 38 | 3 | + | 0.01 | 3434 | None |
| 9 | 20 | 1 | − | n.m. | Unknown | Unknown |
|  | 24 | 2 | + | 0.01 | 2572 | none |

*Parity means the number of times live births in a pregnant mother.

***Abbreviations:*** PVL, proviral load; PBMCs, peripheral blood mononuclear cells; n.m., not measured.

unknown; not investigated
